# A phase 3 study of rituximab biosimilar HLX01 in patients with diffuse large B-cell lymphoma

**DOI:** 10.1186/s13045-020-00871-9

**Published:** 2020-04-16

**Authors:** Yuankai Shi, Yongping Song, Yan Qin, Qingyuan Zhang, Xiaohong Han, Xiaonan Hong, Dong Wang, Wei Li, Yang Zhang, Jifeng Feng, Jianmin Yang, Huilai Zhang, Chuan Jin, Yu Yang, Jianda Hu, Zhao Wang, Zhengming Jin, Hang Su, Huaqing Wang, Haiyan Yang, Weijun Fu, Mingzhi Zhang, Xiaohong Zhang, Yun Chen, Xiaoyan Ke, Li Liu, Ding Yu, Guo’an Chen, Xiuli Wang, Jie Jin, Tao Sun, Xin Du, Ying Cheng, Pingyong Yi, Xielan Zhao, Chaoming Ma, Jiancheng Cheng, Katherine Chai, Alvin Luk, Eugene Liu, Xin Zhang

**Affiliations:** 1 National Cancer Center/National Clinical Research Center for Cancer/Cancer Hospital, Beijing Key Laboratory of Clinical Study on Anticancer Molecular Targeted Drugs, Chinese Academy of Medical Sciences & Peking Union Medical College, Beijing, China; 2 Affiliated Cancer Hospital of Zhengzhou University, Henan Cancer Hospital, Zhengzhou, China; 3 Harbin Medical University Cancer Hospital, Harbin, China; 4 Fudan University Shanghai Cancer Center, Shanghai, China; 5 Daping Hospital, Third Affiliated Hospital of the Army Medical University, Chongqing, China; 6 The First Bethune Hospital of Jilin University, Changchun, China; 7 The Second Hospital of Dalian Medical University, Dalian, China; 8 Jiangsu Cancer Hospital and Jiangsu Institute of Cancer Research, Nanjing, China; 9 Changhai Hospital, Shanghai, China; 10 Tianjin Medical University Cancer Institute & Hospital, Tianjin, China; 11 Cancer Center of Guangzhou Medical University, Guangzhou, China; 12 Fujian Provincial Cancer Hospital, Fuzhou, China; 13 Fujian Medical University Union Hospital, Fuzhou, China; 14 Beijing Friendship Hospital, Capital Medical University, Beijing, China; 15 The First Affiliated Hospital of Soochow University, Soochow, China; 16 The 307th Hospital of Chinese People’s Liberation Army, Beijing, China; 17 Tianjin People’s Hospital, Tianjin, China; 18 Zhejiang Cancer Hospital, Hangzhou, China; 19 Shanghai Changzheng Hospital, Shanghai, China; 20 The First Affiliated Hospital of Zhengzhou University, Zhengzhou, China; 21 The Second Affiliated Hospital of Zhejiang University School of Medicine, Hangzhou, China; 22 Jinan Central Hospital Affiliated to Shandong University, Jinan, China; 23 Peking University Third Hospital, Beijing, China; 24 Tangdu Hospital, Air Force Medical University, Xi’an, China; 25 Hubei Cancer Hospital, Wuhan, China; 26 The First Affiliated Hospital of Nanchang University, Nanchang, China; 27 The Second Hospital of Jilin University, Changchun, China; 28 The First Affiliated Hospital, College of Medicine, Zhejiang University, Hangzhou, China; 29 Liaoning Cancer Hospital & Institute, Shenyang, China; 30 Guangdong Provincial People’s Hospital, Guangzhou, China; 31 Jilin Cancer Hospital, Changchun, China; 32 Hunan Cancer Hospital, Changsha, China; 33 Xiangya Hospital, Central South University, Changsha, China; 34 Shanghai Henlius Biotech, Inc., Shanghai, China

**Keywords:** Rituximab biosimilar, DLBCL, Efficacy equivalence

## Abstract

Rituximab in combination with chemotherapy has shown efficacy in patients with diffuse large B-cell lymphoma (DLBCL) for more than 15 years. HLX01 was developed as the rituximab biosimilar following a stepwise approach to demonstrate biosimilarity in analytical, pre-clinical, and clinical investigations to reference rituximab. With demonstrated pharmacokinetic similarity, a phase 3 multi-center, randomized, parallel, double-blind study (HLX01-NHL03) was subsequently conducted to compare efficacy and safety between HLX01 plus cyclophosphamide, doxorubicin, vincristine, and prednisone (H-CHOP) and reference rituximab plus CHOP (R-CHOP) in a total of 407 treatment-naïve, CD20-positive DLBCL patients aged 18–80 years. The primary efficacy endpoint was best overall response rate (ORR) within six cycles of treatment in the per-protocol set (PPS). Secondary endpoints included 1-year efficacy outcomes, safety, and immunogenicity profile. The results showed difference in ORRs [H-CHOP 94.1%; R-CHOP 92.8%] between two treatment groups was 1.4% (95% confidence interval [CI], − 3.59 to 6.32, *p* = 0.608) which falls within the pre-defined equivalence margin of ± 12%. The safety profile was comparable between the treatment groups, with a similar overall incidence of treatment-emergent adverse events (H-CHOP 99.5%, R-CHOP 99.0%, *p* = 1.000) and serious adverse events (H-CHOP 34.0%, R-CHOP 32.5%, *p* = 0.752). This study established bioequivalence in efficacy and safety between HLX01 and reference rituximab. The trial was registered at http://www.chinadrugtrials.org.cn on 26 August 2015 [#CTR20150583].

Treatment with rituximab, a monoclonal antibody against CD20, in combination with cyclophosphamide, doxorubicin, vincristine, and prednisone (R-CHOP) has been used in patients with diffuse large B-cell lymphoma (DLBCL) for more than 15 years with proven efficacy and safety [1]. With demonstrated highly similar analytical characterization and bioequivalence in pharmacokinetics and pharmacodynamics [2], we conducted this phase 3, multi-center, randomized, parallel, double-blind study (HLX01-NHL03) to establish the equivalence in clinical efficacy, safety, and immunogenicity between HLX01 plus CHOP (H-CHOP) and R-CHOP every 21 days for up to six cycles in treatment-naïve patients with CD20-positive DLBCL.

Eligible patients were treatment-naïve adults (≥ 18 to ≤ 80 years) with International Prognostic Index of 0-2, clinical stages I–IV (Ann Arbor Staging) and histologically confirmed CD20-positive DLBCL. The primary efficacy endpoint was best overall response rate (ORR) within six cycles of treatment in the per-protocol set (PPS), and secondary efficacy endpoints included complete response rate, 1-year duration of response, 1-year event-free survival, 1-year progression-free survival, 1-year disease-free survival, 1-year overall survival, and depletion of CD19-positive B-cells in peripheral blood.

From October 9, 2015 to March 10, 2017, a total of 560 patients were screened, of whom 407 patients were randomized (1:1) at 33 investigational sites; 361 patients (H-CHOP 173; R-CHOP 188) completed six cycles of treatment, and 328 patients (H-CHOP 157; R-CHOP 171) completed the study (Fig. 1a). Baseline characteristics are well balanced between two treatment groups (Fig. 1b). In the PPS, the best ORRs within six cycles of treatment in the PPS were 94·1% (95% confidence interval [CI], 89.77 to 97.04) and 92·8% (95% CI, 88.19 to 96.00) in the H-CHOP and R-CHOP groups, respectively, with an intergroup difference of 1.4% (95% CI, − 3.59 to 6.32, *p* = 0.608). The efficacy equivalence between HLX01 and reference rituximab was demonstrated with 95% CIs falls entirely within the pre-defined margin of ± 12%. The results of using the full analysis set (FAS) were consistent with the primary efficacy analysis in the PPS. Previous reports of R-CHOP in patients with DLBCL have shown ORRs ranging between 83% and 88% [3, 4], which is comparable with the result from this study. No significant differences were observed in the 1-year analysis of all secondary efficacy endpoints, in either the PPS or the FAS (Table 1).

**Fig. 1:**
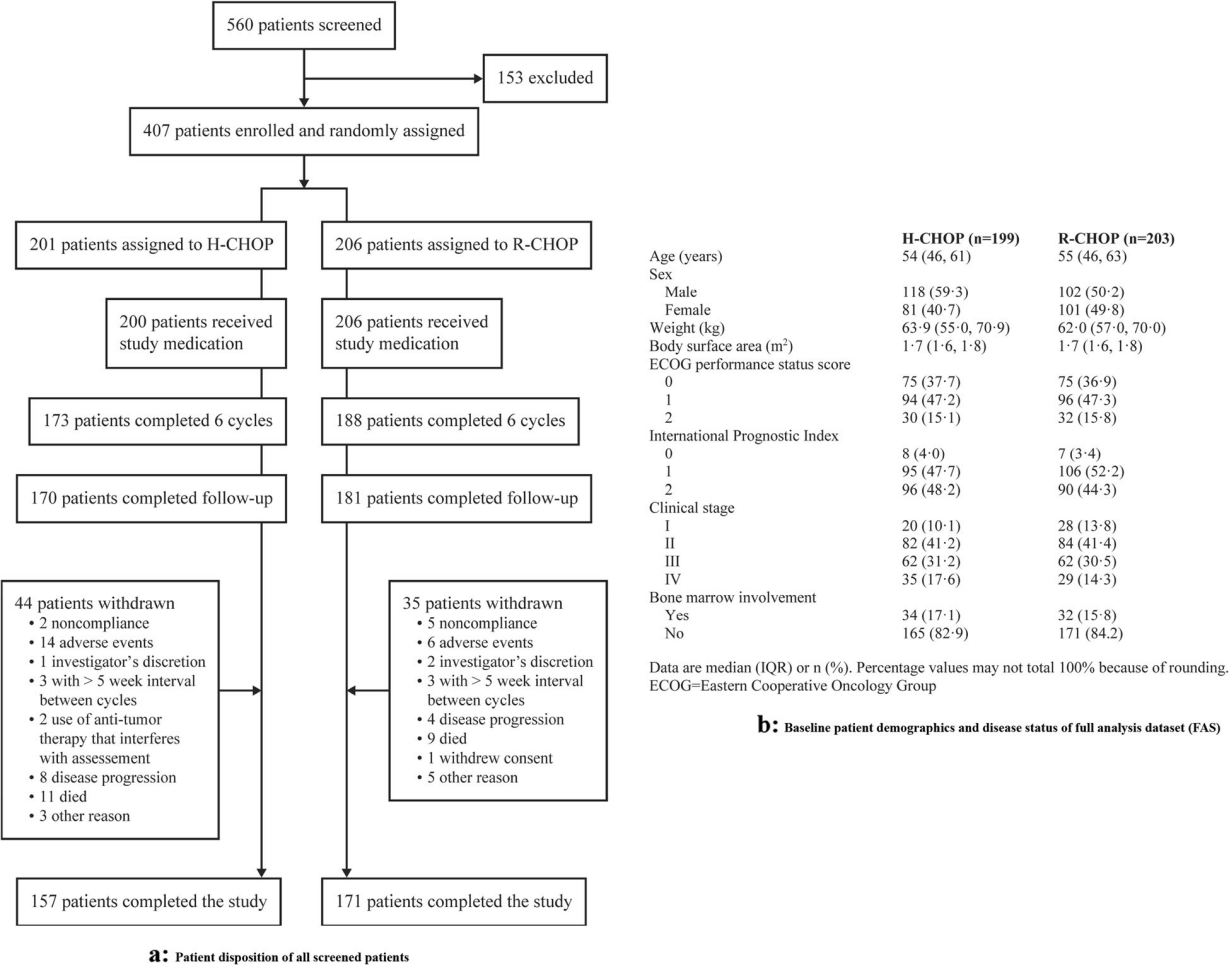
**a** Patient disposition of all screened patients. **b** Baseline patient demographics and disease status of full analysis dataset (FAS)

**Table 1:** Efficacy outcomes

	Per-protocol dataset			Full analysis dataset		
	H-CHOP (***n*** = 188)	R-CHOP (***n*** = 194)	***P*** value	H-CHOP (***n*** = 199)	R-CHOP (***n*** = 203)	***P*** value
Best overall response rate	177 (94·1)	180 (92·8)	0·608	184 (92·5)	187 (92·1)	0·839
Complete response	88 (46·8)	101 (52·1)	0·231	90 (45·2)	104 (51·2)	0·190
Partial response	89 (47·3)	79 (40·7)		94 (47·2)	83 (40·9)	
Stable disease	8 (4·3)	13 (6·7)		11 (5·5)	15 (7·4)	
Disease progression	2 (1·1)	1 (0·5)		2 (1·0)	1 (0·5)	
No evidence of disease	1 (0·5)	0		2 (1·0)	0	
Duration of response						
Patients experiencing events	25 (13·8)	21 (11·5)	0·424	26 (13·7)	21 (11·1)	0·355
Patients censored	156 (86·2)	161 (88·5)		164 (86·3)	168 (88·9)	
Event-free survival						
Patients experiencing events	80 (42·6)	67 (34·5)	0·125	88 (44·2)	71 (35·0)	0·087
Patients censored	108 (57·4)	127 (65·5)		111 (55·8)	132 (65·0)	
1-year event-free survival rate	55·4 (47·9, 63·0)	64·5 (57·6, 71·4)		53·7 (46·4, 61·0)	63·4 (56·6, 70·2)	
Progression-free survival						
Patients experiencing events	31 (16·5)	29 (14·9)	0·534	33 (16·6)	30 (14·8)	0·473
Patients censored	157 (83·5)	165 (85·1)		166 (83·4)	173 (85·2)	
1-year progression-free survival rate	75·0 (66·5, 83·6)	80·1 (73·5, 86·7)		74·1 (65·6, 82·7)	79·7 (73·1, 86·3)	
Overall survival						
Patient deaths	15 (8·0)	13 (6·7)	0·661	16 (8·0)	14 (6·9)	0·701
Patients censored	173 (92·0)	181 (93·3)		183 (92·0)	189 (93·1)	
1-year overall survival rate	91·8 (87·8, 95·8)	92·4 (88·3, 96·6)		91·6 (87·6, 95·5)	92·1 (88·0, 96·3)	
Disease-free survival						
Patients experiencing events	27 (14·4)	24 (12·4)	0·462	28 (14·1)	25 (12·3)	0·477
Patients censored	161 (85·6)	170 (87·6)		171 (85·9)	178 (87·7)	
1-year disease-free survival rate	77·4 (68·9, 85·9)	83·0 (76·7, 89·3)		76·9 (68·4, 85·4)	82·6 (76·2, 88·9)	

Data are n (%) or %, (95% CI). Percentage values may not total 100% because of rounding

The safety analysis set (Table 2) comprised 406 patients who received at least one treatment. 199/200 in H-CHOP group and 204/206 in R-CHOP group (H-CHOP 99.5%, R-CHOP 99.0%, *p* = 1.000) experienced at least one treatment-emergent adverse event; 68/200 in H-CHOP and 67/206 in R-CHOP (H-CHOP 34.0%, R-CHOP 32.5%, *p* = 0.752) experienced at least one serious adverse event; 14/200 in H-CHOP and 9/206 in R-CHOP (H-CHOP 7.0%, R-CHOP 4.4%, *p* = 0.252) discontinued treatment because of adverse events (AEs). The most common AEs were hematological events such as decreased white blood cell count (H-CHOP 85.5%; R-CHOP 85.9%), decreased neutrophil count (H-CHOP 79.0%; R-CHOP 81.6%), and anemia (H-CHOP 38.5%; R-CHOP 35.0%).

**Table 2:** Safety profiles in the safety analysis dataset

	H-CHOP (***n*** = 200)	R-CHOP (***n*** = 206)
Patients with ≥1 TEAE	199 (99·5)	204 (99·0)
Patients with ≥1 SAE	68 (34·0)	67 (32·5)
Patients with ≥1 AE leading to treatment discontinuation	14 (7)	9 (4·4)
Patients deaths due to AE	5 (2·5)	3 (1·5)
Adverse events with an incidence ≥10%		
Hematological		
Decreased white blood cell count	171 (85·5)	177 (85·9)
Decreased neutrophil count	158 (79·0)	168 (81·6)
Anemia	77 (38·5)	72 (35·0)
Decreased platelet count	34 (17·0)	19 (9·2)
Decreased lymphocyte count	24 (12·0)	34 (16·5)
Decreased hemoglobin concentration	23 (11·5)	20 (9·7)
Non-hematological		
Nausea	46 (23·0)	49 (23·8)
Increased alanine aminotransferase	49 (24·5)	38 (18·4)
Fever	47 (23·5)	34 (16·5)
Decreased appetite	32 (16·0)	42 (20·4)
Increased lactate dehydrogenase	30 (15·0)	40 (19·4)
Debilitation	38 (19·0)	31 (15·0)
Alopecia	35 (17·5)	34 (16·5)
Increased aspartate aminotransferase	34 (17·0)	30 (14·6)
Cough	31 (15·5)	26 (12·6)
Vomiting	22 (11·0)	30 (14·6)
Upper respiratory tract infection	19 (9·5)	29 (14·1)
Hypokalemia	28 (14·0)	17 (8·3)
Constipation	27 (13·5)	25 (12·1)
Non-infectious pneumonia	19 (9·5)	24 (11·7)
Pulmonary infection	19 (9·5)	24 (11·7)
Diarrhea	16 (8·0)	22 (10·7)
Chills	20 (10·0)	14 (6·8)
Adverse events by CTCAE Grade		
Grade 1	8 (4·0)	6 (2·9)
Grade 2	35 (17·5)	35 (17·0)
Grade 3	54 (27·0)	75 (36·4)
Grade 4	98 (49·0)	85 (41·3)
Grade 5	4 (2·0)	3 (1·5)
Grade 4 adverse events with an incidence ≥2·5%		
Decreased neutrophil count	85 (42·5)	75 (36·4)
Decreased white blood cell count	44 (22·0)	42 (20·4)
Febrile neutropenia	5 (2·5)	6 (2·9)
Bone marrow failure	5 (2·5)	5 (2·4)

Data are n (%). Percentage values may not total 100% because of rounding

Among the patients observed with infusion-related reactions (IRRs), 61/200 in H-CHOP group and 61/206 in R-CHOP group (H-CHOP 30.5%; R-CHOP 29.6%), the most common reactions were those affecting skin and subcutaneous tissues. Most IRRs were grade 1 or 2, and no grade 4 or 5 IRRs were reported. Increases in hepatitis B virus (HBV) DNA titer were observed in five patients in H-CHOP group and eight patients in R-CHOP group, and nine of whom were receiving antiviral therapy for chronic HBV; however, no patients developed signs or symptoms of fulminant hepatitis.

Anti-drug antibodies (ADAs) were detected in one patient (< 1%) in each treatment group at baseline and immediately before administration of the second treatment cycle. After 6 months of follow-up, ADAs were detected in one patient in H-CHOP group and two patients in R-CHOP group (H-CHOP 1.0%, R-CHOP 1.7%, *p* = 1.000), and after 8 months of follow-up in seven patients in H-CHOP group and six patients in R-CHOP group (H-CHOP 7.1%, R-CHOP 5.5%, *p* = 0.629). During the entire study, only one patient in R-CHOP group had both ADAs and neutralizing antibodies.

In conclusion, this study demonstrated therapeutic equivalence between HLX01 and reference rituximab. The analysis of the primary and secondary efficacy endpoints did not reveal any statistically significant differences between two treatment groups. The safety and immunogenicity profiles of HLX01 were comparable with reference rituximab with no clinically meaningful differences observed between two treatment groups.

## Data Availability

The data that support the findings of this study are available from the corresponding author upon reasonable request.

## Abbreviations

R-CHOP: Rituximab plus cyclophosphamide, hydroxydaunorubicin, oncovin (vincristine), and prednisone
DLBCL: Diffuse large B-Cell lymphoma
H-CHOP: HLX01 plus cyclophosphamide, hydroxydaunorubicin, oncovin (vincristine), and prednisone
ORR: Best overall response rate
PPS: Per-protocol set
CI: Confidence interval
FAS: Full analysis set
AE: Adverse event
IRR: Infusion-related reaction
HBV: Hepatitis B virus
ADA: Anti-drug antibody
